# CCR7 Expression and Intratumoral FOXP3^+^ Regulatory T Cells are Correlated with Overall Survival and Lymph Node Metastasis in Gastric Cancer

**DOI:** 10.1371/journal.pone.0074430

**Published:** 2013-09-05

**Authors:** Shuang Zhou, Shuchang Xu, Huihong Tao, Zhiwei Zhen, Guolin Chen, Zhiqiang Zhang, Yaoqin Yang

**Affiliations:** 1 Department of Histology and Embryology, Tongji University School of Medicine, Shanghai, China; 2 Department of Gastroenterology, Tongji Hospital, Tongji University School of Medicine, Shanghai, China; 3 Department of Preventive Medicine, Tongji University School of Medicine, Shanghai, China; University of South Carolina School of Medicine, United States of America

## Abstract

The aim of this study was to investigate the prognostic value of chemokine receptor CCR7 expression and intratumoral FOXP3^+^ regulatory T cells (Tregs) in gastric cancer. CCR7^+^ tumor cells and FOXP3^+^ Tregs were assessed by immunohistochemistry in tissue microarrays containing gastric cancer from 133 patients. Prognostic effects of low or high CCR7 and FOXP3 expression were evaluated by Cox regression and Kaplan-Meier analysis, as well as the correlation between CCR7 positive score and intratumoral FOXP3^+^ cell number in a longitudinal assessment. The analysis showed that the high expression levels of CCR7 and FOXP3 were detected in 69.9% and 65.4% of cases, respectively. High CCR7 expression in gastric cancer cells was significantly associated with poor overall survival (OS) (*P* = 0.010) and lymph node metastasis (*P* = 0.009), and was an independent factor for worse OS (*P* = 0.023) by multivariate analysis. High numbers of intratumoral FOXP3^+^ Tregs significantly correlated with shorter OS (*P* = 0.021) and lymph node metastasis (*P* = 0.024), and was also an independent factor for adverse OS (*P* = 0.035). Furthermore, there was a significantly positive correlation between CCR7 positive score and intratumoral FOXP3^+^ cell number (r = 0.949, *P*<0.001). These results revealed that CCR7 expression in gastric cancer cells and intratumoral FOXP3^+^ Tregs could be considered as a co-indicator of clinical prognosis of gastric cancer.

## Introduction

CCR7 is a seven transmembrane G protein-coupled chemokine receptor, mainly expressed on the cell surface of naive T cells and dendritic cells (DCs), SLC (secondary lymphoid chemokine, CCL21) and ELC (EB11-ligand chemokine, CCL19) are its two high-affinity ligands. CCR7 is essential for the migration of T cells and DCs to the surrounding lymphoid tissue or the site of immune response by binding to its ligands [1]. *CCR7* is *expressed not only* in naive T cells and DCs, but also in some tumor cells. Tumors with high CCR7 expression are more likely to exhibit extrathyroidal extension, angiolymphatic invasion, lymph node metastasis, anti-apotosis and cell proliferation [2]–[4]. CCR7 promotes tumor growth and metastasis in response to endogenous SLC or ELC. Many studies have shown that malignant cells with CCR7 expression can be directed to the corresponding organs with high presence of their ligands [5]–[8]. Therefore, CCR7-mediated cell chemotaxis is not only related to the establishment of the antitumor immune microenvironment, but also may help the early evaluation of the biological behavior of tumors. CCR7 expression in tumor cells is closely correlated with their metastasis and malignancy [2].

Regulatory T cells (Tregs) are believed to dampen T-cell immunity and to be the main obstacle tempering immunotherapy. A growing body of evidence suggests that Tregs within the tumor microenvironment might play a significant role in the suppression of local antitumor immune responses [9], [10]. FOXP3, a forkhead/winged helix transcription factor, is the most specific marker of Tregs and it is possible to define Tregs more strictly as CD4^+^ CD25^+^ regulatory T cells [11]. FOXP3 appears to be critical for the development and function of Tregs, and the loss of FOXP3 expression leads to the lack of Tregs and hyperactivation of CD4^+^ T cells, resulting in lethal autoaggressive lymphoproliferation, whereas overexpression of FOXP3 results in severe immunodeficiency [11]–[13]. It is reported that increased FOXP3^+^ Tregs are associated with a higher risk of recurrence and poor overall survival of patients with some solid neoplasms [14]–[20].

Gastric cancer is the fourth most common cancer in the world, and the overall survival of patients remains poor despite improved diagnostic and treatment strategies, among which resection is one of the first priorities [21]. Further investigation is recommended to examine the prognostic impact of CCR7 expression and Tregs in gastric cancer.

In this study, we investigated the prognostic value of CCR7 expression in gastric cancer cells and intratumoral FOXP3^+^ Tregs, and the relationship between them in gastric cancer. The results suggested that they were not only associated with poor survival rates and lymph node metastasis, but also positively correlated with each other.

## Materials and Methods

### Ethics Statement

This study was approved by the ethical committee at Tongji University School of Medicine and written informed consent was obtained from all patients before enrollment. The procedure of collecting human gastric cancer resection specimens from patients was conformed to the principles outlined in the Declaration of Helsinki.

### Patients and Follow-up

133 patients with gastric cancer who underwent extended lymph node dissection (D2 resection) between 2001 and 2007 at Tongji Hospital (Shanghai, China) were enrolled in our study. The evaluation of resection specimens was performed in accordance with the guidelines of the Japanese Gastric Cancer Association (JGCA) in 1998. None of the patients received radiotherapy, chemotherapy or other medical interventions before the surgery. Specimens were selected only on the basis of the availability of suitable formalin-fixed, paraffin-embedded tissue and complete clinicopathologic and follow-up data for the patients.

Follow-up was completed on 15 November, 2010. A minimum 3-year follow-up was required. The median follow-up was 43 months (range 36 to 104 months). Follow-up procedures consisted of interim history, physical examination, tumor markers (CEA, CA199), abdominal ultrasonography and X-ray every 4–6 months according to the postoperative time. For patients with test results suggestive of recurrence, computed tomography (CT) and magnetic resonance imaging (MRI) were used for corroborative evidence of relapse. The recurrences of gastric cancer had to be confirmed by cytology biopsy or surgery. Overall survival (OS) was defined as the interval between surgery and death or between surgery and the last observation for surviving patients. The data were censored at the last follow-up for living patients.

### Tissue Microarrays and Immunohistochemistry

Tissue microarrays were constructed as previously described [22], [23]. All cases were histologically reviewed by haematoxylin and eosin staining, and representative tumor areas with small round lymphocyte infiltrate were premarked in the paraffin blocks, away from necrotic and haemorrhagic materials. Duplicate 1-mm diameter cylinders from tumor centre (designated as intratumor) were selected from each case using an automated tissue arrayer (Shanghai Biochip Co., Ltd, Shanghai, China), together with paracancerous nonmalignant gastric tissues as controls to ensure reproducibility and homogenous staining of the slides. Sections of 4-µm thickness were taken on 3-aminopropyltriethoxysilane-coated slides.

The mouse monoclonal antibodies used were anti-human CCR7 (eBioScience, San Diego, CA) and FOXP3 (Biolegend, San Diego, CA). Immunohistochemistry of paraffin sections was carried out using a two-step protocol (Novolink Polymer Detection System, Novocastra, Newcastle, UK) according to the manufacturer’s instructions. Briefly, sections were deparaffinized in xylene and rehydrated in ethanol. After endogenous peroxidase activity was blocked with incubation of the slides in 0.3% H_2_O_2_, antigen retrieval was done by placing the sections in an electric kitchen pot filled with boiling citrate buffer for 20 min, and nonspecific binding sites were blocked with Protein Block (RE7102). After serial incubation with primary antibodies, Post Primary Block (RE7111), and secondary antibodies (RE7112), the sections were developed in 3,3′-diamiobenzidine solution under a microscope and counterstained with hematoxyline. Negative control slides omitting the primary antibodies were included in all assays.

### Evaluation of Immunohistochemical Variables

The CCR7 positive score of tumor cells was analyzed by use of image analysis software (Leica Qwin Image Processing and Analysis Application Software, Wetzlar, Germany). For each slide, all intact high-power field (HPF) (×400) digital images were taken by the aforementioned equipment and then analyzed by the image analysis software to acquire the positive score of CCR7 for each field. Finally, average number for one HPF (×400) was calculated to ensure the representativeness and homogeneity.

The number of FOXP3^+^ Tregs in microarrays was counted as previously described [14], using computerized image analysis system composed of a Hitachi HV-C20A CCD camera (Hitachi, Tokyo, Japan), installed on a Leica DMLA light microscope (Leica Microsystems, Wetzlar, Germany) and attached to a personal computer. Under ×400 magnification, there were at least 12 independent and intact computerized microscopic fields for the duplicates of each patient sample. 8 independent microscopic fields (×400), representing the densest lymphocytic infiltrates, were selected for each patient sample to ensure representativeness and homogeneity. The numbers of 12 fields were cumulated and then averaged to calculate the final number for one computerized ×400 microscopic field (0.0768 mm^2^/field). The evaluation of FOXP3^+^ Tregs was performed by two independent observers in a blinded fashion. Discrepancies in enumeration, within a range of 5%, were re-evaluated and a consensus decision was made.

### Statistical Analysis

Actuarial OS rates were calculated by the Kaplan-Meier method and analyzed by the log-rank test. Univariate and multivariate analyses were based on the Cox proportional hazards regression model. A secondary analysis was performed to assess the relationship among variables and clinicopathologic characteristics. For the comparison of individual variables, chi-square tests and Fisher’s exact tests were carried out as appropriate. Two-tailed *P*<0.05 was judged to be significant. All analyses were performed using SPSS 16.0 software (SPSS, Chicago, IL).

## Results

### Patient Characteristics

The characteristics of 133 patients were shown in Table 1. The samples included 89 (66.9%) male and 44 (33.1%) female patients with gastric cancer. 68 (51.1%) patients were less than 60 year-old and others were more than or equal to 60 year-old. The tumor size of 79 (59.4%) patients was less than 40 mm and that of others was more than or equal to 40 mm. 75 (56.4%) patients had lymphatic invasion and 84 (63.2%) patients had lymph node metastasis. 61 (45.9%) tumors were undifferentiated and others were differentiated. 50 (37.6%) tumors were categorized as T1T2, and 83 (62.4%) as T3T4 according to JGCA.

**Table 1:** The characteristics of 133 patients with gastric cancer.

Characteristics	No. of patients	%
Gender		
Male	89	66.9
Female	44	33.1
Age (years)		
<60	68	51.1
≥60	65	48.9
Tumor size (mm)		
<40	79	59.4
≥40	54	40.6
Lymphatic invasion		
Negative	58	43.6
Positive	75	56.4
Lymph node metastasis		
Negative	49	36.8
Positive	84	63.2
Histological type		
Undifferentiated	61	45.9
Differentiated	72	54.1
T Classification		
T1T2	50	37.6
T3T4	83	62.4

### Expression of CCR7 and FOXP3 in Gastric Cancer Tissues

**Figure 1:**
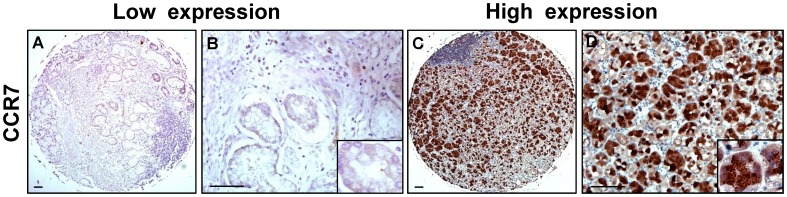
Expression of CCR7 in tissue microarrays of gastric cancer. (**A**–**D**) Representative immunostainings of low and high CCR7 expression. CCR7 positive cells were identified in the cytoplasm and cell membrane of gastric cancer cells. Scale bar, 200 and 50 µm.

**Figure 2:**
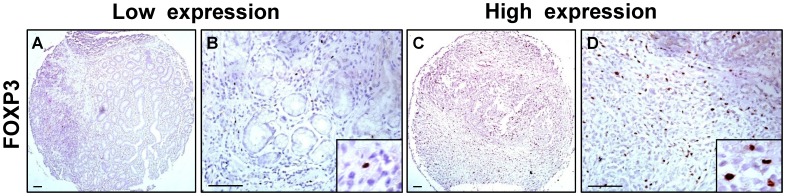
Expression of FOXP3 in tissue microarrays of gastric cancer. (**A**–**D**) Representative immunostainings of low and high FOXP3 expression. Cells were considered as FOXP3^+^ Tregs based on distinct intranuclear expression. Scale bar, 200 and 50 µm.

The expression levels of CCR7 and FOXP3 in tissue microarrays of gastric cancer were examined by immunohistochemical analysis. Fig. 1 and Fig. 2 showed representative immunostainings of CCR7 and FOXP3. Positive staining of CCR7 was identified in the cytoplasm and cell membrane of gastric cancer cells (Fig. 1 A–D). High CCR7 expression was detected in 69.9% of gastric cancer samples (93 cases of 133 patients). Cells were considered as FOXP3^+^ Tregs based on distinct intranuclear expression (Fig. 2 A–D). High FOXP3 expression was detected in 65.4% of gastric cancer samples (87 cases of 133 patients). In terms of the score of CCR7 positive tumor cells and FOXP3^+^ Tregs number, the statistical results for the immunohistochemical variables were shown in Table 2. As the controls, there were no CCR7 expression and very few FOXP3^+^ Tregs in normal gastric tissues (Fig. S1).

**Table 2:** Descriptive statistics of immunohistochemical variables.

Variables	Mean	SE	Range
FOXP3^+^ cell number	9.85	0.92	0–78.3
CCR7 positive score	15.32	1.35	0–86.9

### CCR7 and FOXP3 Expression were Associated with Overall Survival in Gastric Cancer

At last follow-up, 39 patients (29.3%) studied had died, all of whom died from the recurrence of gastric cancer. The OS rates were 93.5% for 1 year, 81.2% for 2 years and 76.9% for 3 years, respectively, for the whole study.

**Figure 3:**
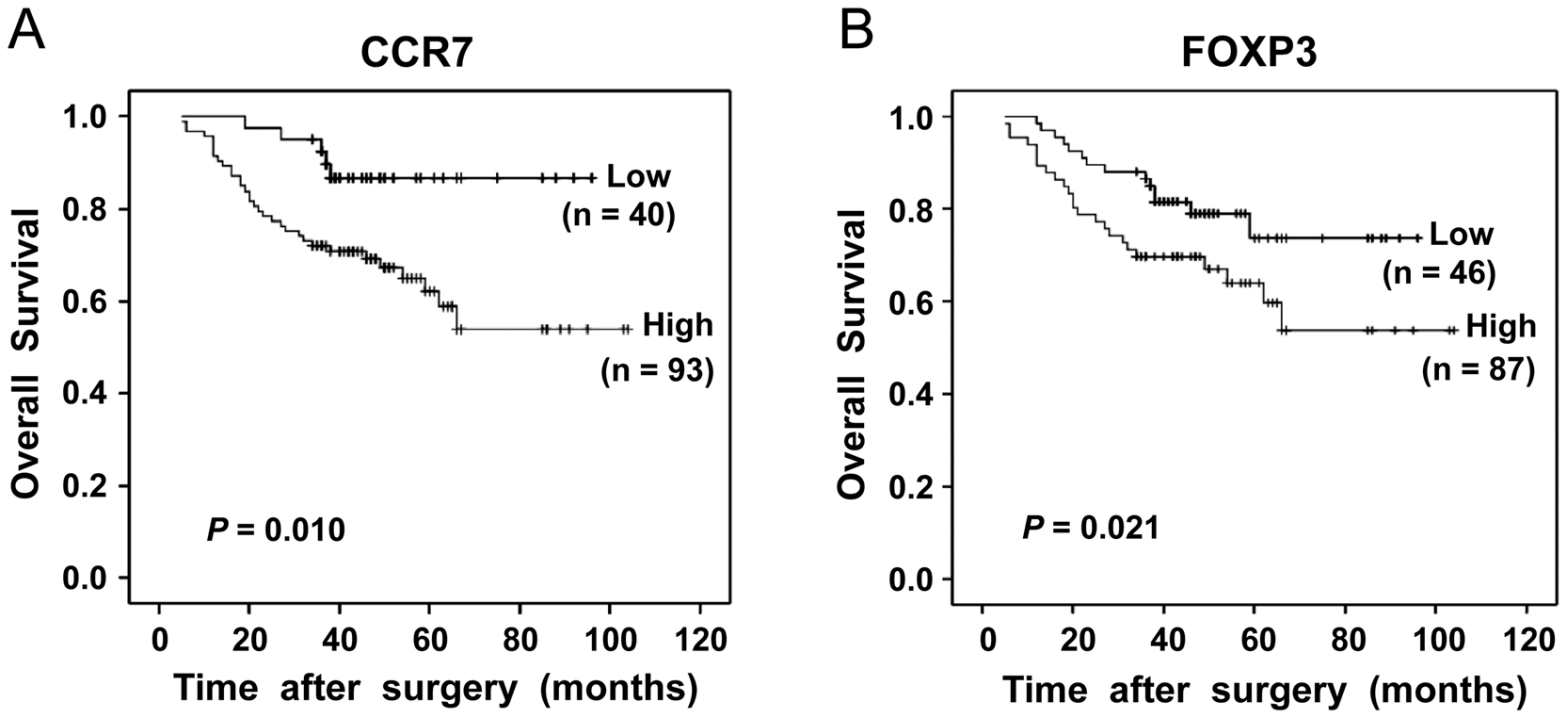
Kaplan-Meier analysis of overall survival in patients for CCR7 and FOXP3 expression after surgery. (**A**) High CCR7 expression in gastric cancer cells was associated with poor overall survival (log-rank test, *P* = 0.010); (**B**) High numbers of intratumoral FOXP3^+^ Tregs were also associated with worse overall survival (log-rank test, *P = *0.021).

Prognostic effects of low or high CCR7 and FOXP3 expression were evaluated by Cox regression and Kaplan-Meier analysis. On univariate analysis, age, gender, tumor size and tumor differentiation showed no prognostic significance for the OS rates, but lymphatic invasion, lymph nodes metastasis and tumor (T) classification were associated with the OS rates (*P* = 0.019, *P* = 0.013, *P*<0.001, respectively, Table 3). CCR7 expression in gastric cancer cells was associated with the OS rates (log-rank test, *P* = 0.010<0.05), one-year, two-year and three-year OS rates were 91.4%, 78.5% and 72% for the high CCR7 expression groups, respectively, compared with 100%, 97.5% and 92.4% for the low CCR7 expression groups (Fig. 3A). Multivariate analysis showed that high CCR7 expression was an independent prognostic factor for worse OS rates (hazard ratio [HR] 2.896, 95% confidential interval [CI] 1.385–7.276, *P* = 0.023<0.05, Table 4). High infiltration of intratumoral FOXP3^+^ Tregs was also associated with adverse OS (log-rank test, *P* = 0.021<0.05), one-year, two-year and three-year OS rates were 88.9%, 79.1% and 69.5% for the high FOXP3 expression groups, respectively, compared with 97.8%, 89.6% and 87.2% for the low FOXP3 expression groups (Fig. 3B). On multivariate analysis, the presence of high intratumoral FOXP3^+^ Tregs was also showed to be an independent predictor for worse OS rates (HR 1.906, 95% CI 1.205–4.238, *P* = 0.035<0.05, Table 5).

**Table 3:** Univariate analysis of factors associated with overall survival.

Variables	Overall Survival		
	HR	95%CI	*P*
Age (years) (<60 v≥60)	0.685	0.396–1.324	0.307
Gender (Male v Female)	0.543	0.287–1.035	0.121
Tumor Size (mm) (<40 v≥40)	1.126	0.631–2.057	0.405
Lymphatic invasion (negative v positive)	2.675	1.582–5.403	0.019
Lymph node metastasis (negative v positive)	2.983	2.147–7.685	0.013
Differentiation (Yes v No)	1.329	0.854–3.216	0.178
T Classification (T1T2 v T3T4)	6.521	2.723–15.616	<0.001
CCR7 expression (low v high)	3.226	1.258–8.267	0.015
Foxp3 expression (low v high)	2.403	1.187–4.521	0.026

**Table 4:** Multiple analysis of CCR7 expression associated with overall survival.

Variables	Overall Survival		
	HR	95%CI	*P*
Lymphatic invasion (negative v positive)	2.025	1.073–4.626	0.031
Lymph node metastasis (negative v positive)	2.298	1.412–6.355	0.025
T Classification (T1T2 v T3T4)	5.284	1.968–12.031	0.008
CCR7 expression (low v high)	2.896	1.385–7.276	0.023

**Table 5:** Multiple analysis of FOXP3 expression associated with overall survival.

Variables	Overall Survival		
	HR	95%CI	*P*
Lymphatic invasion (negative v positive)	1.974	1.052–4.109	0.033
Lymph node metastasis (negative v positive)	2.135	1.215–4.927	0.029
T Classification (T1T2 v T3T4)	4.681	1.429–10.852	0.011
FOXP3 expression (low v high)	1.906	1.205–4.238	0.035

### Associations between the Expression Levels of CCR7 and FOXP3 and Clinical Characteristics

Table 6 summarized the relationship between the expression levels of CCR7 and FOXP3 and clinical characteristics of the 133 patients with gastric cancer. Both CCR7 and FOXP3 expression were significantly correlated with lymph node metastasis (*P* = 0.009 and 0.024, respectively). There was also a significant association between CCR7 expression and lymphatic invasion (*P* = 0.012). However, the statistically analysis showed that there was no significant association between CCR7 and FOXP3 expression and other clinical characteristics, such as gender, age, tumor size, differentiation and T classification.

**Table 6:** Associations between the expression levels of CCR7 and FOXP3 and clinical characteristics.

Characteristics	FOXP3 expression			CCR7 expression		
	Low	High	*P*	Low	High	*P*
Gender						
Male	36	53	0.057	29	60	0.078
Female	10	34		11	33	
Age (years)						
<60	22	46	0.325	18	50	0.402
≥60	24	41		22	43	
Tumor size (mm)						
<40	26	53	0.183	22	57	0.115
≥40	20	34		18	36	
Lymphatic invasion						
Negative	27	31	0.059	30	28	0.012
Positive	19	56		10	65	
Lymph node metastasis						
Negative	34	15	0.024	33	16	0.009
Positive	12	72		7	77	
Histological type						
Undifferentiated	25	36	0.195	26	35	0.167
Differentiated	21	51		14	58	
T Classification						
T1T2	22	28	0.153	17	33	0.441
T3T4	24	59		23	60	

### Correlation between CCR7 Positive Score and Intratumoral FOXP3^+^ Cell Number

**Figure 4:**
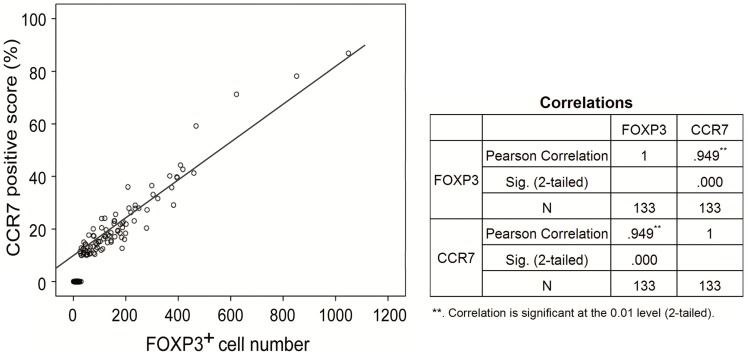
The correlation between CCR7 positive score and intratumoral FOXP3^+^ cell number. There was a significantly positive correlation between CCR7 positive score of gastric cancer cells and intratumoral FOXP3^+^ Treg cell number (r = 0.949, *P*<0.001) in a longitudinal assessment.

The correlation between CCR7 positive score and intratumoral FOXP3^+^ Treg cell number was evaluated in a longitudinal assessment. CCR7 positive score of gastric cancer cells and intratumoral FOXP3^+^ Treg cell number could be represented in the same scatterplot, from the scatterplot, two variables seemed to have a linear trend that could be carried out linear regression analysis. And then fitting two variables in applied statistics linear correlation analysis was performed to further analyze their correlation. The result showed that there was a significantly positive correlation between CCR7 positive score of gastric cancer cells and intratumoral FOXP3^+^ Treg cell number (r = 0.949, *P*<0.001, Figure 4).

## Discussion

In the process of tumor occurrence and development, CCR7 on the tumor cell surface binding to its ligand SLC can promote tumor cell proliferation, angiogenesis in the tumor tissue, and directional migration [24]. SLC/CCR7 complex can cause cells to induce actin polymerization, which is closely correlated with pseudopod formation of the tumor cell and necessary for its invasion and metastasis [25]. Previous studies have found that high expression level of CCR7 in lung cancer, esophageal carcinoma, hepatocellular cancer, breast cancer and melanoma closely related to tumor invasion and lymph node metastasis [5]–[7], [26]–[28]. Thus, whether CCR7 expression or not and the level of its expression may directly affect cancer metastasis and prognosis. CCR7 expression in some tumor cells even can be used as molecular markers to determine whether the tumors are *metastatic* [29]. However, these findings and other issues such as prognostic factors need further research in vivo and clinical confirmation.

The nuclear transcription factor FOXP3 has been widely accepted as the best marker for Tregs identification in humans thus far. The expression of FOXP3 in T cells corresponds with immune regulatory function in the tumor microenvironment [30]. Tumor-infiltrating FOXP3^+^ Tregs were reported to be increased in a variety of tumors [31]–[36]. A few studies have also investigated the clinicopathologic significance of Tregs infiltration [32]–[34]. Marked infiltration of Tregs in cancer stroma was found to be an unfavorable prognostic factor in liver [14], [20], ovarian [17], breast [19], tonsillar [32], pancreatic [33] and colon [34] cancers. So far as we know, whether FOXP3^+^ Tregs are an unfavorable predictor for gastric cancer is still controversial [37]–[39].

In this study, we explored whether CCR7 expression in gastric cancer cells and intratumoral FOXP3^+^ Tregs were associated with clinicopathological factors and could be used as a prognostic indicator. In tissue microarrays of gastric cancers collected from 133 patients, which have been shown to be a powerful tool for evaluating tumor specimens [40], we used an immunohistochemical method to examine the expression levels of CCR7 and FOXP3 in gastric cancer tissues. High CCR7 expression was detected in 69.9% of 133 gastric cancer samples. The result was consistent with the earlier study of Mashino et al., who have used RT-PCR to detect 64 cases of gastric cancer tissue samples, of which 68.8% of gastric cancer tissues are CCR7 mRNA positive expression [25]. Importantly, our results indicated that CCR7 expression in gastric cancer cells was associated with OS by Kaplan-Meier analysis, patients with high CCR7 expression tumors had a significantly shorter survival than those with low CCR7 expression tumors. Furthermore, multivariate analysis demonstrated that CCR7 expression was an independent prognostic indicator in gastric cancer. In our series, similarly, using Kaplan-Meier analysis, the results showed that the prevalence of high intratumoral FOXP3^+^ Tregs was significantly related to poor survival rates of patients with gastric cancer. Multivariate analysis indicated that intratumoral FOXP3^+^ Tregs could be also an independent prognostic factor for patients with gastric cancer. Therefore, our study demonstrated that both high CCR7 expression and increased intratumoral FOXP3^+^ Tregs could be considered as an indicator of poor prognosis in gastric cancer.

Increasing evidence shows that CCR7 may play an important role in directing tumor cells to the draining lymph nodes and contribute to the frequent presence of lymph node metastasis in cancer patients [7]–[9]. The chemotactic SLC/CCR7 interaction may be a possible mechanism for induction by cancer cells of lymph node metastasis and tissue invasion. This hypothesis was supported by our findings that the high expression of CCR7 was significantly correlated with the presence of lymph node metastasis and lymphatic invasion. The association between the expression level of FOXP3 and clinical characteristics of the 133 patients with gastric cancer was also included in the present study. It was worth noting that both CCR7 and FOXP3 expression were significantly correlated with lymph node metastasis. Nextly, we performed linear correlation analysis to find a significantly positive correlation between CCR7 positive score and intratumoral FOXP3^+^ Treg cell number. These findings demonstrated that the high expression of CCR7 in gastric cancer cells and large amount of intratumoral FOXP3^+^ Tregs infiltration might play a critical role in lymph node metastasis, and therefore influence the treatment outcomes of gastric cancer.

Our study confirmed the importance of CCR7 expression in gastric cancer cells and intratumoral FOXP3^+^ Tregs as prognostic factors, which were correlated with overall survival and lymph node metastasis in gastric cancer, in line with the previous findings [25], [38]. In the present study, it was interesting that either CCR7 expression or intratumoral FOXP3^+^ Tregs was found to be an independent prognostic factor in gastric cancer. This is, to the best of our knowledge, the first report demonstrating that high CCR7 expression in gastric cancer cells was in parallel with marked infiltration of intratumoral FOXP3^+^ Tregs in the tumor microenvironment. Further studies will be needed to understand whether blockade of SLC/CCR7 interaction in combination with FOXP3^+^ Tregs depletion can inhibit the metastasis of gastric cancer cells to lymph nodes, and lead to better treatment outcomes or not.

In conclusion, our results demonstrated that both high CCR7 expression in tumor cells and increased intratumoral FOXP3^+^ Tregs were associated with worse OS in gastric cancer, positively correlated with each other, and could be considered as a co-indicator of clinical prognosis of gastric cancer. Based on this, developing molecular target therapy against CCR7 and FOXP3 might be a promising strategy for cancer treatment.

## Supporting Information

Figure S1 **Expression of CCR7 and FOXP3 in normal gastric tissues.** Representative immunostainings for CCR7 and FOXP3. As the controls, there were no CCR7 expression and very few FOXP3^+^ Tregs in normal gastric tissues. Scale bar, 50 µm.
(TIFF)
