# Correction: CCR7 Expression and Intratumoral FOXP3^+^ Regulatory T Cells are Correlated with Overall Survival and Lymph Node Metastasis in Gastric Cancer

**DOI:** 10.1371/journal.pone.0097056

**Published:** 2014-05-05

**Authors:** The PLOS ONE Staff

Some of the work reported in this article was conducted by researchers based at the department of Anatomy, Histology and Embryology, Shanghai Medical College, Fudan University. The authors from Fudan University should have been included in the author list in the article, the authors apologize for this omission.

The author list should be corrected to read as below:

Shuang Zhou^1,3☯^, Zhenbing Shen^2☯^, Yanna Wang^2^, Huiying Ma^1^, Shuchang Xu^4^, Jie Qin^1^, Long Chen^1^, Huihong Tao^3^, Zhiwei Zhen^3^, Guolin Chen^3^, Zhiqiang Zhang^5^, Rilun Li^1^, Honglei Xiao^1^, Cuiping Zhong^1^, Yaoqin Yang^3^*, Chunmin Liang^1^*

^1^ Lab of Tumor Immunology, Department of Anatomy and Histology & Embryology, Shanghai Medical College of Fudan University, Shanghai, China.

^2^ The General Surgery Department of Zhongshan Hospital, Fudan University, Shanghai, China.

^3^ Department of Histology and Embryology, Tongji University School of Medicine, Shanghai, China.

^4^ Department of Gastroenterology, Tongji Hospital, Tongji University School of Medicine, Shanghai, China.

^5^ Department of Preventive Medicine, Tongji University School of Medicine, Shanghai, China.

^☯^These authors equally contributed to this work. * Corresponding author: Chunmin Liang, cmliang@fudan.edu.cn, Principle Investigator of the Lab of Tumor Immunology, Department of Anatomy and Histology & Embryology, Shanghai Medical College, Fudan University, 138 Yixueyuan Road, 200032, Shanghai, China; Yaoqin Yang, yaoqiny@163.com, Department of Histology and Embryology, Tongji University School of Medicine, 1239 Siping Road, 200092, Shanghai, China.

The authors from Fudan University declare that no competing interests exist, their contributions to the study are as below:

Conceived and designed the experiments: Chunmin Liang

Performed the immunohistochemistry: Zhenbing Shen, Huiying Ma, Jie Qin, Honglei Xiao

Performed quantitation experiments: Long Chen, Rilun Li

Analyzed the data: Chunmin Liang, Zhenbing Shen, Yanna Wang

Contributed reagents/materials/analysis tools: Cuiping Zhong, Chunmin Liang

Contributed to the design of the experiments and the writing of the manuscript: Cuiping Zhong

In addition the authors would like to acknowledge Guomin Zhou and Yihong Sun for their help in providing materials to this work.

The funding statements should also be corrected to acknowledge the grants that supported the authors from Fudan University:

This work was supported by grants from the National Science Foundation of China (No.30871312, No. 31000527), the National Basic Research Program of China (973-program, No.2011CB910700), Shanghai Health Bureau Research Fund for young investigator (to Dr. Shuang Zhou) and Hong Kong Scholar program (No. XJ2011025).
