# Supplementary figures and images for: CCR7 Expression and Intratumoral FOXP3+ Regulatory T Cells are Correlated with Overall Survival and Lymph Node Metastasis in Gastric Cancer
Source: PLoS One. 2013 Sep 5;8(9):e74430. doi: 10.1371/journal.pone.0074430 (PMC3764061; doi:10.1371/journal.pone.0074430)

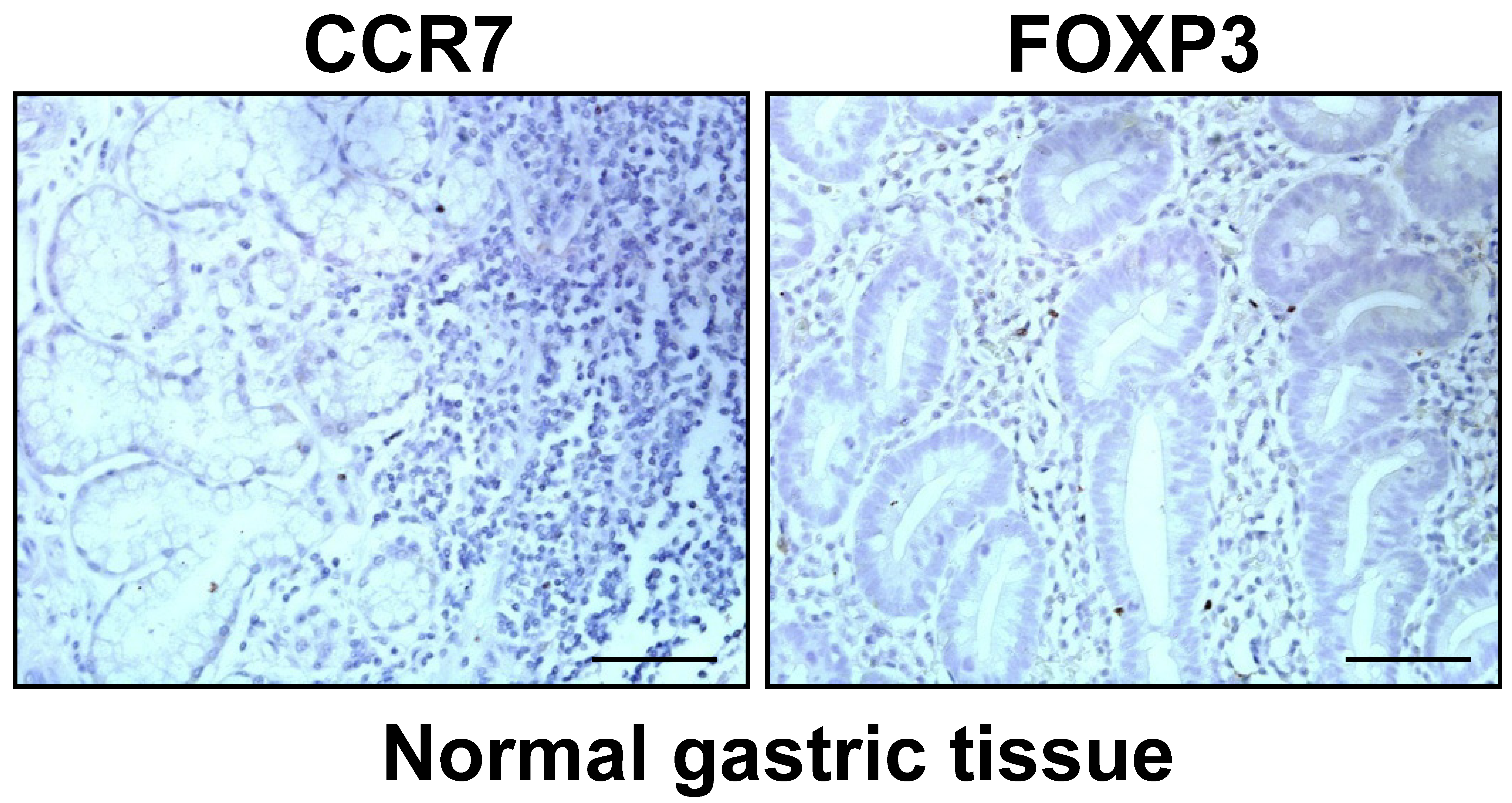

Supplement: Figure S1 — Expression of CCR7 and FOXP3 in normal gastric tissues. Representative immunostainings for CCR7 and FOXP3. As the controls, there were no CCR7 expression and very few FOXP3+ Tregs in normal gastric tissues. Scale bar, 50 µm. (TIFF) [file pone.0074430.s001.tif]
